# Editorial: Unravelling Social Norm Effects: How and When Social Norms Affect Eating Behavior

**DOI:** 10.3389/fpsyg.2018.00738

**Published:** 2018-05-15

**Authors:** F. Marijn Stok, Saar Mollen, Kirsten T. Verkooijen, Britta Renner

**Affiliations:** ^1^Department of Interdisciplinary Social Science, Utrecht University, Utrecht, Netherlands; ^2^Department of Psychology, University of Konstanz, Konstanz, Germany; ^3^Amsterdam School of Communication Research, University of Amsterdam, Amsterdam, Netherlands; ^4^Department of Social Sciences, Wageningen University and Research Center, Wageningen, Netherlands

**Keywords:** social norms, eating behavior, descriptive norms, injunctive norms, social influence, moderator effects

## Purpose of the research topic

A consistent body of research has shown that human eating behavior is affected by the social environment. For example, it has been shown that obesity spreads through social networks (Christakis and Fowler, 2007) and that the presence of others substantially affects people's food intake (Herman et al., 2003; Cruwys et al., 2015). A key aspect of the social environment are the social norms that exist in a group of people (Cialdini, 2003). Seminal studies on social norms (e.g., Asch, 1951; Goldstein et al., 2008) have shown that providing individuals with information about what other group members think or do (i.e., *descriptive social norms*) or what other group members expect them to do (i.e., *injunctive social norms*) can influence these individuals' thoughts and behaviors.

Social norms have also been found to affect eating behavior (for reviews, see Robinson et al., 2014b; Higgs, 2015; Stok et al., 2016), and social norms have become the focal point of a wide range of interventions aimed at changing unhealthy eating behavior (e.g., Stok et al., 2012; Mollen et al., 2013a; Robinson et al., 2014a). However, the outcomes of social norm interventions for health promotion have been mixed, with positive effects (Turner et al., 2008), no effects (Granfield, 2005), and even counterproductive effects being reported (Clapp et al., 2003). The variation in outcomes suggests that social norm effects are not yet well-understood. Indeed, we still know relatively little about how and when the so-called “social norms approach” is effective (Burchell et al., 2013), and what are the moderating and mediating variables in the context of eating behavior.

Because of the great potential that social norms hold as a tool for improving eating behavior, improved insight into social norms' working mechanisms is urgently needed. The purpose of this Research Topic was therefore to increase our understanding of social norm effects in the context of eating behavior, focusing specifically on research examining mediators and/or moderators of social norm effects on eating behavior. The large number of views since opening the research topic (20,369 views for Research Topic on 2-2-18) suggests that the relevance of the topic is widely recognized by the scholarly community.

## Overview of contributions

Most studies in the current Research Topic focused on the consumption of unhealthy foods and/or drinks (Giese et al.; Hirata et al.; Wansink and Kniffin; Jones and Robinson), while relatively little attention was paid to the consumption of healthy foods (only in Thomas et al.). Most studies the sample consisted of university students. There were two exceptions; Giese et al. investigated a large sample of children and adolescents in three different countries, and Versluis and Papies used a consumer panel in their second study. A strength of studies in the current topic is that they mostly focused on actual eating behavior as the outcome measure, either self-reported (Giese et al.; Jones and Robinson) or objectively measured (Hirata et al.; Thomas et al.; Wansink and Kniffin).

Most studies investigated *when* social norms influence eating behavior (i.e., effect moderators). Factors that were investigated were the type of norm (descriptive norm vs. liking norm; Thomas et al.), habit strength (Thomas et al.), identification with the group (Versluis and Papies), individual food preferences (Giese et al.), self-control (Jones and Robinson), presence of others (Wansink and Kniffin), time (Thomas et al.; Jones and Robinson), and self-esteem (Giese et al.; Hirata et al.). Only one study investigated *why* social norms affect eating behavior (i.e., effect mediators): Versluis and Papies investigated whether perceptions about the appropriateness of portion sizes mediated the relationship between norms and expected portion sizes.

Positive self-concept or self-esteem was the focus of two studies (Giese et al.; Hirata et al.). While Giese et al. found that children and adolescents conformed to a social norm more when they had positive self-concept, this was not confirmed in the students investigated by Hirata et al. One explanation for these diverging findings may lie in the source of social influence under study: while Giese et al. studied how group level healthy vs. unhealthy food preferences influenced individual snack intake, Hirata et al. examined to what extent food consumption was matched in female dyads. Another explanation may lie in the operationalization of self-concept: Giese et al. measured social self-esteem (e.g., “I am popular”), while Hirata et al. considered self-esteem aspects related to individual characteristics (e.g., “I have good qualities”). Future research should investigate whether the importance of one's self-concept in conformity to social norms depends on the source of the social norm, but also which parts of a person's self-concept are especially relevant in the domain of social influence. Another avenue worthy of future research is the causal direction of this relationship. While a positive social self-concept may mean that one values the group and therefore adheres to group norms, it may also be the case that conforming to group norms serves to enhance one's self-concept (Cialdini and Goldstein, 2004).

Time effects also sparked the interest of multiple researchers (Thomas et al.; Jones and Robinson). Jones and Robinson showed that perceived descriptive norms predicted self-reported intake of cakes and pastries 1 year later (although the effect was small), but not sugar sweetened beverages or alcohol. On the other hand, Thomas et al. found that broccoli consumption (but not consumption of other foods) was higher in low habitual vegetable consumers after they heard that a majority of students likes vegetables a lot, irrespective of whether eating behavior was assessed immediately or a day later. These studies provide some indication that social norms may affect eating behaviors immediately, but also at a later point in time. The strength with which norms affect behavior over time and situations may, however, depend on the type of norm under consideration. Prior research has found for instance that injunctive, but not descriptive, norm messages affected behavior 1 month later (Mollen et al., 2013b), while another previous study did show that descriptive (majority vs. minority) norm messages had an effect on behavior across a period of a week (Stok et al., 2014). More research on longitudinal effects of social norms is deemed necessary.

While most studies investigated the moderators of social norm effects on eating behavior, only one study attempted to gain more insight into the mediators of social norm effects. Versluis and Papies found that the amount of food that was thought to be appropriate to serve oneself partially mediated the relationship between a portion size norm and the amount people expected to serve themselves. Extant literature ties different motives for conformity to different types of norms. While the influence of descriptive norms on behavior is frequently tied to informational motives, i.e., the desire to make an accurate choice, injunctive social norms are frequently tied to normative motives, i.e., the desire to affiliate (Deutsch and Gerard, 1955). The study by Versluis and Papies shows that, in the domain of eating behavior, there is indeed evidence that information about what others deem appropriate (akin to injunctive norms) affects one's perception about what is the appropriate course of action, which then partially explains people's expected behavior.

## Conclusion

The aim of this research topic was to explore the mechanisms (the “how” and “when”) underlying the social norm effect in the context of eating behavior. The large diversity in contributions illustrates the wide application, and hence possible implications, of the topic. Overall, the present studies did not yield evidence of strong mediators or moderators of the social norm effect on eating behavior, and showed a tendency to investigate moderators more than mediators. Nevertheless, the results provide important leads for further research into potential relevant moderators, like self-esteem, habit strength as well as mediators, like individual (norm) perceptions. Recommendations for future research would be to more frequently investigate healthy food consumption, to include more non-student populations, to pay more attention to longer-term effects of social norms on eating behavior, and to focus more on mediation effects.

## Author contributions

All authors were part of the editorial team of the research topic, edited manuscripts, and reviewed manuscripts. FS, SM, and KV wrote the first version of the editorial. BR extensively revised the editorial. All authors agree to submission of the editorial for publication in its current version.

### Conflict of interest statement

The authors declare that the research was conducted in the absence of any commercial or financial relationships that could be construed as a potential conflict of interest.
